# Supplementary material for: Application of High-Throughput Sequencing for Comprehensive Virome Profiling in Grapevines Shows Yellows in Iran
Source: Viruses. 2024 Jan 29;16(2):204. doi: 10.3390/v16020204 (PMC10891595; doi:10.3390/v16020204)
Supplement: Supplementary file 1 [file viruses-16-00204-s001.zip › viruses-2826173-supplementary.pdf]

Table S1. List of reference sequences used for mapping of individual viruses and viroids

Virus/viroid	abbreviation	GenBank accession number
Garlic virus A	GarV-A	JX997952
Papaya mosaic virus	PapMV	MH645156
Apple stem pitting virus	ASPV	NC003462
Grapevine Pinot gris virus	GPGV	NC015782
Grapevine virus A	GVA	DQ855088
Alfalfa mosaic virus - RNA1	AMV	Mk913782
Cucumber mosaic virus – RNA2	CMV	LC604033
Ambrosia asymptomatic virus 2	AAV2	NC055542
Grapevine leafroll associated virus 1	GLRaV1	NC016509
Grapevine leafroll associated virus 3	GLRaV3	KY764333
Grapevine endophyte endornavirus	GEEV	JX678977
Grapevine red blotch virus	GRBV	NC022002
Iranian johnsongrass mosaic virus	IJMV	JQ692088
Turnip mosaic virus	TuMV	KJ936089
Arabis mosaic virus RNA1	ArMV	NC006057
RNA2		NC006056
Grapevine deformation virus RNA1	GDefV	NC017939
RNA2		NC017938
Grapevine fanleaf virus RNA1	GFLV	KU522584
RNA2		KU522585
Physalis rugose mosaic virus	PhyRMV	MK681145
Grapevine Red Globe virus	GRGV	NC030693
Grapevine fleck virus	GFKV	AJ309022
Grapevine satellite virus	GV-Sat	NC021480
Citrus exocortis viroid	CEVd	DQ318794
Grapevine yellow speckle viroid 1	GYSVd1	NC001920
Grapevine yellow speckle viroid 2	GYSVd2	KJ489020
Australian grapevine viroid	AGVd	NC003553
Hop stunt viroid	HSVd	NC001351

Table S2. List of primer pairs used for the detection of viruses in grapevine libraries

Virus	Primer name	amplicon	Annealing temp		Primer seq	Ref
Australian grapevine viroid	H1(+)<br>C1(-)	300	59	Forward<br>Reverse	GTCGACGAAGGGTCCTCAGCAGAGCACC<br>CTCGACGACGAGTCGCCAGGTGAGTCTT	[38]
Hop stunt viroid	HSV+<br>HSV-	300	59	Forward<br>Reverse	GGCAACTCTTCTCAGAATCCAGC<br>CCGGGGCTCCTTTCTCAGGTAAGT	[38]
Grapevine yellow speckle viroid 1	GV2(+)<br>GV1(-)	300	60	Forward<br>Reverse	TAAGAGGTCTCCGGATCTTCTTGC<br>GCGGGGGTTCCGGGGATTGC	[39]
Grapevine leafroll-associated virus 1	LEV1-C447<br>LQV1-H47	398	56	Forward<br>Reverse	CGACCCCTTTATTGTTTGAGTATG<br>GTTACGGCCCTTTGTTTATTATGG	[40]
Grapevine fanleaf virus	DetF<br>DetR	1000	56	Forward<br>Reverse	CGGCAGACTGGCAAGCTGT<br>GGTCCAGTTTAATTGCCATCCA	[23]
Grapevine deformation virus	GDefV-F<br>GDefV-R	371	55	Forward<br>Reverse	GCCCAGGATGAGCTCTACTAC<br>TATGGGGAATTGTGTGGCCA	[41]
Arabis mosaic virus	ArMV-F<br>ArMV-R	1518	58	Forward<br>Reverse	AATCCTTTTGGAATGCCAGGCAATCC<br>ACAAGCTAAATATAAATATAATACA	[42]
Grapevine red blotch virus	GRBV-V2F<br>GRBV-V1R	798	56	Forward<br>Reverse	ATGGGTTAGGGGATGAGGCT<br>CGGCAATGACTCCTGCGGCT	[43]
GVA	GVA-F<br>GVA-R	240	54	Forward<br>Reverse	AGGTCCACGTTTGCTAAG<br>CATCGTCTGAGGTTTCTA	[44]
Alfalfa mosaic virus	AMV-F<br>AMV-R	351	58	Forward<br>Reverse	CCATCATGAGTTCTTCACAAAAG<br>TCGTCACGTCATCAGTGAGAC	[45]
Garlic virus A	GVA-F<br>GVA-R	417	55	Forward<br>Reverse	TGGAGACCCTTTCCAAGGCA<br>CTCATCCGCGAATGGTGGTT	[46]

Table S3. Comparison of efficiency of three software packages in reconstruction of viral/viroidal genomes from sRNA sequences of grapevine libraries generated by high-throughput sequencing

Virus/viroid	Reference GenBank accession No.	Reference genome Size (nt)	CLC workbench		UGENE package		VIRUSDETECT pipeline	
			Genome recovery (nt)	Genome coverage (%)	Genome recovery (nt)	Genome coverage (%)	Genome recovery (nt)	Genome coverage (%)
Apple stem pitting virus	NC003462	9332	1697	18.18	9247	99.09	nf	0
Physalis rugose mosaic virus	MK681145	4175	606	14.51	4152	99.45	nf	0
Papaya mosaic virus	MH645156	6866	991	14.43	6837	99.58	nf	0
Turnip mosaic virus	KJ936089	9834	1371	13.94	9714	98.78	nf	0
Grapevine virus A	DQ855088	7342	1013	13.80	7296	99.37	nf	0
Cucumber mosaic virus – RNA2	LC604033	3018	386	12.79	3006	99.63	nf	0
Grapevine fleck virus	AJ309022	7564	952	12.60	7552	99.84	414	5.5
Ambrosia asymptomatic virus 2	NC055542	7408	901	12.16	7381	99.64	nf	0
Grapevine leafroll associated virus 3	KY764333	18449	2173	11.78	18249	98.92	nf	0
Grapevine endophyte endornavirus	JX678977	12154	1340	11.00	12005	98.77	nf	0
Iranian johnsongrass mosaic virus	JQ692088	9544	961	10	9331	97.77	nf	0

Table S4. Description and frequency in the virome of viruses and viroids identified in five Iranian grapevine cultivars by sRNA sequencing and confirmed by PCR from composite samples of grapevine leaves collected at the commercial vineyards in northeast of Iran

Family	Genus	Virus/viroid Species	Cultivar	Peykani		Askari-Bidaneh		Peykani		Rezghi		Sahebi		Fakhri	
			Library	A4		A5		A6		A7		A8		A9	
			Abbreviation	NGS	PCR	NGS	PCR	NGS	PCR	NGS	PCR	NGS	PCR	NGS	PCR
Alphaflexiviridae	Potexvirus	Papaya mosaic virus	PapMV	nf	nt	nf	nt	0.17	nt	nf	nt	nf	nt	nf	nt
Betaflexiviridae	Foveavirus	Apple stem pitting virus	ASPV	nf	nt	nf	nt	nf	nt	nf	nt	nf	nt	1.86	nt
	Vitivirus	Grapevine virus A	GVA	nf	nt	nf	nt	nf	nt	nf	nt	nf	nt	0.53	nt
Bromoviridae	Cucumovirus	Cucumber mosaic virus	CMV	0.62	nt	2.86	nt	nf	nt	nf	nt	nf	nt	nf	nt
Caulimoviridae	Badnavirus	Ambrosia asymptomatic virus 2	AAV2	nf	nt	0.95	nt	3.88	nt	4.16	nt	0.64	nt	nf	nt
Closteroviridae	Ampelovirus	Grapevine leafroll associated virus 3	GLRaV3	nf	nt	nf	nt	nf	nt	nf	nt	nf	nt	2.39	nt
Endornaviridae	Endornavirus	Grapevine endophyte endornavirus	GEEV	1.26	nt	nf	nt	nf	nt	nf	nt	nf	nt	nf	nt
Potyviridae	Potyvirus	Iranian johnsongrass mosaic virus	IJMV	nf	nt	nf	nt	nf	nt	0.27	nt	nf	nt	nf	nt
		Turnip mosaic virus	TuMV	nf	nt	nf	nt	0.17	nt	nf	nt	1.98	nt	nf	nt
Solemoviridae	Sobemovirus	Physalis rugose mosaic virus	PhyRMV	0.31	nt	nf	nt	nf	nt	nf	nt	nf	nt	nf	nt
Tymoviridae	Maculavirus	Grapevine fleck virus	GFKV	nf	nt	nf	nt	0.17	+	nf	nt	nf	nt	nf	nt

nf: not found; nt: not test
